# Bidirectional regulation over the development and expression of loss of control over cocaine intake by the anterior insula

**DOI:** 10.1007/s00213-017-4593-x

**Published:** 2017-04-05

**Authors:** Jean-Yves Rotge, Paul J Cocker, Marie-Laure Daniel, Aude Belin-Rauscent, Barry J Everitt, David Belin

**Affiliations:** 1 AP-HP, Groupe Hospitalier Pitié-Salpêtrière, Service de Psychiatrie d’Adultes, Paris, France; 2 Inserm, CNRS, APHP, Institut du Cerveau et de la Moelle (ICM), Hôpital Pitié-Salpêtrière, Sorbonne Universités, UPMC Univ Paris 06, 75013 Paris, France; 3 Department of Psychology, University of Cambridge, Downing Street, Cambridge, CB2 3EB UK

**Keywords:** Addiction, Anterior insula, Cocaine, Escalation

## Abstract

**Rationale:**

Increasing evidence suggests that the anterior insular cortex (AIC) plays a major role in cocaine addiction, being implicated in both impaired insight and associated decision-making and also craving and relapse. However, the nature of the involvement of the insula in the development and maintenance of cocaine addiction remains unknown, thereby limiting our understanding of its causal role in addiction. We therefore investigated whether pre- and post-training bilateral lesions of the AIC differentially influenced the development and the expression of the escalation of cocaine self-administration during extended access to the drug.

**Methods:**

In a series of experiments, Sprague Dawley rats received bilateral excitotoxic lesions of the AIC either prior to, or after 3 weeks of training under 12-h extended self-administration conditions, which are known to promote a robust escalation of intake. We also investigated the influence of AIC lesions on anxiety, as measured in an elevated plus maze and sensitivity to conditioned stimuli (CS)- or drug-induced reinstatement of an extinguished instrumental response.

**Results:**

Whereas, post-escalation lesions of the AIC, as anticipated, restored control over cocaine intake and prevented drug-induced reinstatement, pre-training lesions resulted in a facilitation of the development of loss of control with no influence over the acquisition of cocaine self-administration or anxiety.

**Conclusions:**

AIC lesions differentially affect the development and maintenance of the loss of control over cocaine intake, suggesting that the nature of the contribution of cocaine-associated interoceptive mechanisms changes over the course of escalation and may represent an important component of addiction.

## Introduction

The anterior insular cortex (AIC) has been shown to be involved in the maintenance of drug addiction (Naqvi and Bechara 2010). In humans, the AIC is activated during craving (Bonson et al. 2002; Brody et al. 2002; Naqvi et al. 2014), whilst unilateral lesion of brain regions encompassing the AIC results in sudden loss of the subjective experience of an urge to smoke, thereby facilitating smoking cessation (Abdolahi et al. 2015; Craig 2009; Naqvi and Bechara 2010; Naqvi et al. 2007; Noel et al. 2013). Because of its contribution to the representation of interoceptive states and associated subjective awareness (Craig 2009), the role of the insula in drug addiction has been suggested to be related to maladaptive interoceptive control over behaviour (Naqvi and Bechara 2010; Stewart et al. 2014; Verdejo-Garcia and Bechara 2009; Verdejo-Garcia et al. 2012) and the associated impaired insight (Goldstein et al. 2009; Zaki et al. 2012).

The insula is a heterogeneous integrative cortical region that receives both environmental and internal information in order to generate a cohesive ‘interoceptive representation’ (Craig 2002). The anatomical and cytoarchitectonic organisation of the insula suggests a posterior-to-anterior organisation of information integration (Allen et al. 1991), whereby interoceptive sensory inputs processed within the posterior insula are integrated with emotional evaluative processes within the AIC to produce subjective ‘feelings’ (Zaki et al. 2012).

Through its projections to the prefrontal cortex, the amygdala and the ventral striatal nodes of the corticostriatal circuitry, the AIC influences executive functions and reward-related behaviour both in humans and rats (Belin-Rauscent et al. 2015; Reynolds and Zahm 2005). In rodents, the AIC has been associated with, or shown to be causally involved in, appetitive conditioning (Li et al. 2013; Wu et al. 2014), decision-making (Cocker et al. 2016), impulsivity and the associated vulnerability to develop compulsive behaviour (Belin-Rauscent et al. 2015). Similarly, lesions or inactivations of the AIC influence nicotine intake, cocaine seeking and responsivity to cocaine-related cues (Cosme et al. 2015; Pelloux et al. 2013; Pushparaj et al. 2015), suggesting a role for insula-mediated interoceptive mechanisms in the reinforcing and incentive properties of addictive drugs.

However, the influence of interoceptive mechanisms is not static throughout the course of drug exposure. For example, cocaine methiodide, a quaternary cocaine analogue that does not cross the blood-brain barrier, only gains reinforcing properties in rats with a history of cocaine self-administration (Wang et al. 2013; Wise et al. 2008). This is in agreement with evidence that the insula appears to undergo considerable structural and functional plasticity as a consequence of drug exposure. For instance, the connectivity between the insula and the striatum has been shown to be reduced in drug users (McHugh et al. 2013). Additionally, stimulant users demonstrated lower AIC activation during decision-making in comparison with healthy controls and reduced grey matter volume, which correlates with the duration of drug use (Luo et al. 2013; Mackey and Paulus 2013; Sinha 2011; Stewart et al. 2014).

These data suggest that AIC mechanisms contribute to the regulation of drug-related behaviour, but may be differentially recruited during the development and maintenance of addictive behaviours. Therefore, animals received bilateral excitotoxic lesions of the AIC prior, or subsequent to, acquiring cocaine self-administration in order to investigate any differential role for the AIC during the development and the expression of the escalation of cocaine self-administration, a measure of loss of control over drug use, a key feature of substance use disorder (American Psychiatric Association 2013).

## Materials and methods

### Subjects

Subjects were 48 Sprague Dawley male rats (Charles River, Arbresle, France) weighing ∼250 g at the start of testing. All animals were pair-housed in a climate-controlled colony maintained at 22 ± 1 ^°^C on a reverse light/dark schedule (lights off 7 a.m.). After a week of habituation to the vivarium during which they were handled daily, all rats were food restricted to 85–90% of their free-feeding weight and maintained on 20 g rat chow given daily. Water was available ad libitum. All housing and testing were in accordance with the European Community Directives (2010/63/EU) and were approved by local animal care and use committee.

### Experimental overview

**Fig. 1:**
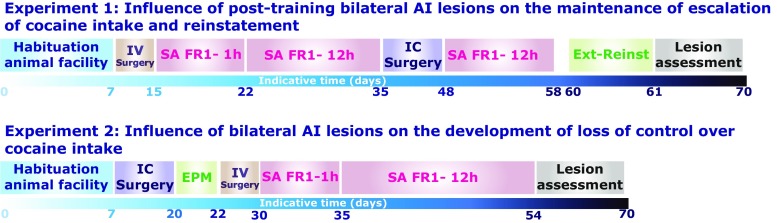
Timeline of the experiments. All rats were habituated to the animal facility for a week during which they were handled and weighed daily. In experiment 1, rats subsequently underwent intrajugular catheterisation (IV surgery), and, after a week of recovery, were trained to acquire cocaine self-administration (SA) over seven daily 1-h sessions under Fixed ratio 1 schedule of reinforcement (FR1). Subsequently, the duration of daily sessions was increased to 12 h wherein all task parameters remained the same. Rats in experiment 1 were exposed to 13 of these sessions, e.g. until a robust and sustained escalation of intake was observed, and subsequently received either sham or bilateral excitotoxic lesions of the AIC. After at least 10 days of recovery, they were then trained again for eight 8 days under extended access conditions prior to be tested in an extinction reinstatement procedure. In experiment 2, rats received either sham or bilateral excitotoxic lesions of the AIC and were given at least 10 days of postoperative recovery prior to be tested for their anxiety in the elevated plus maze (EPM). Rats subsequently underwent intrajugular catheterisation and, after a week of recovery, were trained to acquire cocaine SA over five 1-h daily sessions under FR1. They were then exposed to 19 days of extended 12-h self-administration sessions

The timeline of the two independent experiments is represented Fig. 1. In experiment 1, rats received bilateral or sham lesions of the AIC after repeated extended access sessions, i.e. once loss of control over drug taking had developed and reached an increase in daily infusions of about 50% (as previously shown by Wee et al. 2007 under the same conditions), in order to investigate the involvement of the AIC on the maintenance of escalated cocaine self-administration. Subsequently, the propensity to reinstate extinguished instrumental performance in response to contingent presentation of conditioned stimuli (CS) or non-contingent drug infusions was examined. In experiment 2, rats received either sham or bilateral lesions of the AIC prior to drug exposure. Rats were initially tested on the elevated plus maze in order to measure the influence of AIC manipulation on anxiety, which has previously been shown to predict an increased propensity to escalate cocaine intake (Dilleen et al. 2012). All rats were trained to acquire cocaine self-administration over (5) 1-h sessions and were then exposed to 12-h extended access sessions for 19 days. Twenty-four hours following the last session, rats in each group were sacrificed by perfusion and the brains were harvested for subsequent assessment of the lesions.

Four rats died as a result of complications arising from intracranial or intrajugular surgery, and four did not finish the behavioural training because of catheter failure. Of these, six were from group 1 (6 lesion) and two from group 2 (1 lesion and 1 sham). Additionally, four rats in experiment 2 had a lesion that extended into the adjacent orbitofrontal cortex and were excluded from the final analyses. Therefore, the total number of animals included in all analyses was 36: 16 sham and 6 lesioned for experiment 2, and 7 sham and 7 lesioned for experiment 1.

### Drugs

Cocaine hydrochloride (Cooper, Melun, France) was dissolved in 0.9% sterile saline and the dose calculated as the salt.

### Surgery

Rats were deeply anaesthetised using 2% isoflurane in O_2_ and secured in a stereotaxic frame (World Precision Instruments, FL, USA) with the incisor bar set to −3.3 mm (Paxinos and Watson 1998). Bilateral lesions of the AIC were performed by infusing quinolinic acid (0.8 μl of 0.09 M) infused using a duel-channel infusion pump (Harvard Apparatus, Holliston, MA) at the following coordinates: anteroposterior + 1.44 mm, mediolateral ± 5.2 mm and dorsoventral −6.8 mm (Paxinos and Watson 1998). Infusions took place over 2 min and 30 s. Following infusions, a cannula was left in place for an additional 2 min to ensure adequate diffusion from the injection site. Sham animals underwent the same procedure with the exception that nothing was infused. All animals were allowed to recover for at least 10 days following surgery before testing resumed.

Intrajugular surgical procedures were carried out as previously described (Belin et al. 2011; Belin and Everitt 2008).The rats were anaesthetised with ketamine (100 mg/kg) and xylazine (1 mg/kg), and a Silastic catheter was implanted in the right jugular vein. The proximal end was placed in the right atrium and the distal end passed under the skin and fixed between the scapulae. Animals received prophylactic antibiotics (Baytril 10 mg/kg) administered subcutaneously 1 day prior to, and 6 days following surgery. Animals were returned to their home cage and allowed to recover for 7 days. During this time, catheters were flushed daily with a saline solution containing heparin (20 IU/ml).

### Cocaine self-administration

All self-administration sessions took place in 24 standard self-administration chambers (Med Associates, VT, USA). Each chamber had two retractable levers along one wall located 5 cm above a grid floor. A cue light was located above each lever and the chambers could be illuminated by a houselight. Each chamber was located within a ventilated, sound-attenuating box. During self-administration sessions, the indwelling catheters were attached to a metal spring-covered swivel, connected to a Razel infusion pump (Semat Technical, Herts, UK). Levers were permanently designated as either active or inactive (right/left counterbalanced between animals). Responses on the active lever under a fixed ratio 1 (FR1) schedule of reinforcement lead to an infusion of cocaine (250 μg/50 μl) followed by a 20-s timeout period during which the houselight was switched off, both levers were retracted and a cue light was illuminated above the corresponding lever. Responses on the inactive lever were recorded but had no programmed consequences.

All rats initially acquired cocaine self-administration over several 1-h short access daily sessions and were then exposed to 12-h extended access sessions for several weeks (see Fig. 1), conditions known to promote a robust escalation of cocaine intake (Wee et al. 2007).

### Extinction and reinstatement

Three days after the last self-administration session, rats in experiment 1 were tested during a single session of 210 min consisting of an extinction phase followed by four 30-min CS- or drug-induced reinstatement blocks, as previously described (Deroche-Gamonet et al. 2004). Extinction consisted of a 90-min period during which both active and inactive levers were presented but pressing on either had no programmed consequences. This was immediately followed by a 30-min CS-induced reinstatement test, at the onset of which the cocaine-paired CS were presented non-contingently for 20 s and then for 2 s upon each active lever press, but no cocaine was delivered. This was followed by three non-contingent infusions of increasing doses of cocaine 0.4, 0.8 and 1.6 mg/kg delivered every 30 min during which lever pressing had no programmed consequences. Instrumental performance in response to CS or drug presentation decreased over the second half of each 30-min block reaching levels of responding similar to those observed at the end of the 90-min extinction period. Thus, only the first 15 min of each reinstatement block was compared to the last 15 min of the 90-min extinction period to assess the sensitivity to CS- or drug-induced reinstatement of the extinguished instrumental response, as previously described (Belin et al. 2009).

### Anxiety

Trait anxiety has been shown to predict an increased propensity to escalate cocaine self-administration (Dilleen et al. 2012) and the insula has broadly been implicated in anxiety in both human and animal studies (Alvarez et al. 2015; Li et al. 2014). Therefore, we investigated whether the potential influence of bilateral AIC lesions over escalation of cocaine self-administration may be related to an alteration of anxiety levels. Anxiety was measured on an elevated plus maze as previously described (Vanhille et al. 2015). The maze consisted of four 45 × 10 cm arms, two open and two closed (walls 45 cm high) extending from a central 10 × 10 cm platform located 1 m above the ground. During test sessions, animals were placed on the central platform, facing an open arm and allowed to explore freely the maze for 5 min. Exploration was recorded through an automated tracking system (ViewPoint, Lyon, France). An arm entry was defined as all four paws entering an arm. The number of open arm entries relative to the total number of arm entries along with the percentage of time animals spent in the open arm compared to total time in both arms were calculated to provide a measure of anxiety-like behaviour.

### Lesion assessment

Following completion of all behavioural testing, animals were deeply anaesthetised with a ketamine/xylacine mixture and were transcardiacally perfused with saline (5 min) and 4% formaldehyde (10 min). The brains were harvested and placed in 4% formaldehyde prior to being transferred to a 20% sucrose solution for at least 24 h. The brains were then sectioned in 40-μm coronal sections using a cryostat. Sections were stained with cresyl violet for visualisation of lesion placement and extent.

### Data and statistical analyses

All data are presented as mean ± SEM.

The propensity of the rats to escalate cocaine intake was measured by the escalation ratio calculated as the ratio of drug infusions received on each day relative to the number of infusions received on the first extended access session, which provided a metric of the daily increase in cocaine intake.

The normality of the different distributions was assessed with the Kolmogorov-Smirnov test, and the assumption for homogeneity of variance was tested with the Cochran *C* test.

Data were analysed using Statistica 9 (Dell, OK, USA) and using analysis of variance (ANOVA) with sessions or minutes as within-subject factors and group (lesioned vs sham) as between-subject factor. Analyses for groups 1 and 2 were conducted separately.

For all analyses, upon confirmation of significant main effects, differences among individual means were analysed using the Newman-Keuls post-hoc test. For all analyses, significance was accepted at *α* = 0.05. Effect sizes are reported using partial *η* ^2^ (pη^2^) values (Murray et al. 2015).

## Results

**Fig. 2:**
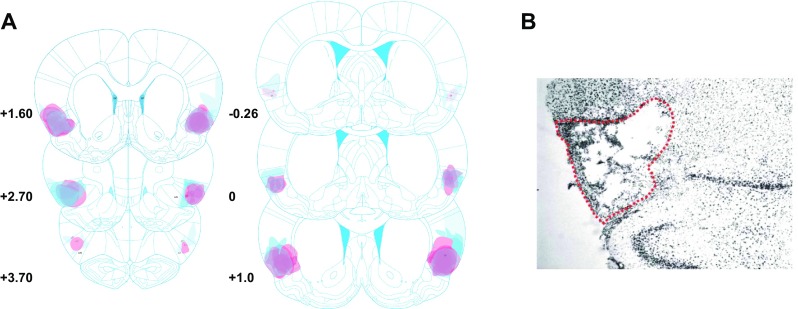
Schematic and histological illustration of bilateral anterior insular lesions. **a** Histological analysis of the extent of bilateral excitotoxic AIC lesions of rats included in experiment 1 (*pink shades*) and experiment 2 (*blue shades*). The extent of the lesions was similar between the two independent experiments. Coordinates are relative to the bregma, plates modified from (Paxinos and Watson 1998). **b** A cresyl violet-stained coronal section of a left AIC lesion (magnification ×6). *Red dotted line* indicates the lesion boundaries

The location and extent of bilateral excitotoxic lesions of the AIC are represented in Fig. 2. Only animals with bilateral and symmetrical lesions of the AIC were included in subsequent analysis.

All rats displayed a robust escalation in cocaine self-administration during extended access sessions [main effect of session: *F*_12,144_ = 44.17, *p* < .001, pη^2^ = .78]. The rats were then split into two groups matched for the rate and magnitude of their escalation and received either sham or AIC lesion surgery [group *F*_1,12_ < 1 and group x session interaction *F*_12,144_ < 1].

**Fig. 3:**
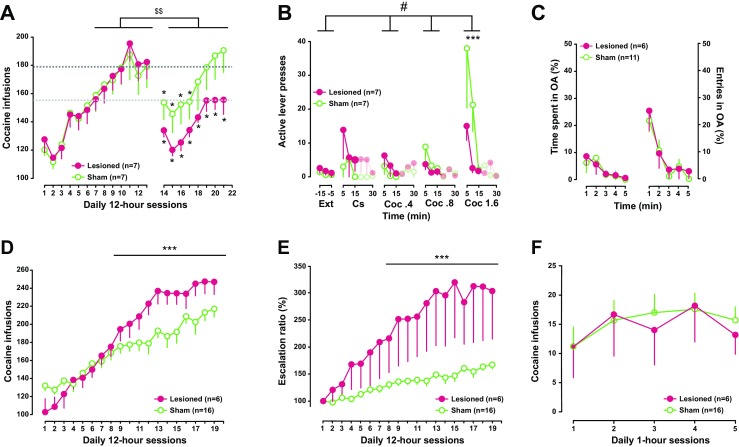
Bidirectional influence of anterior insular lesions on the development and the maintenance of escalated cocaine self-administration. **a** Rats exposed to 12-h daily cocaine self-administration sessions displayed a robust increase in cocaine infusion over time reflecting a loss of control over drug intake [*F*_12,144_ = 44.17, *p* < .001]. Following sham or bilateral AIC excitotoxic lesions, unlike sham-operated rats that quickly returned to pre-surgery levels, lesioned rats showed a long-lasting decrease in their daily intake, indicative of a restoration of control over cocaine self-administration [* indicates significant (*p* < 0.05) difference from last pre-surgery day of long access; $$ indicates main effect of surgery group x pre- and post-surgery block interaction *F*_1,12_ = 7.96, *p* < .02, pη^2^ = .398]. **b** AIC lesions did not influence instrumental performance under extinction. Likewise, lesioned rats did not differ from sham controls in response to Cs-induced reinstatement of instrumental responding. In contrast, AIC lesions prevented the dose-dependent increase in instrumental responding observed in controls in the first 15 min following non-contingent administration of cocaine [m﻿ain effect of group x block interaction F3,36 = 3.36, p < .03, pη2 = .218] [# indicates main effect of group x block interaction; *** indicates *p* < 0.001, post-hoc Newman-Keuls pairwise comparison at the highest dose of cocaine]. **c** AIC lesion did not influence anxiety as measured as the percentage of time spent, or entries, in the open arms of an elevated plus maze over the course of a 5-min test. **d**–**e** In contrast to the restoration of control seen in animals following post-training lesions, pre-training AIC lesions facilitated and exacerbated escalation of cocaine intake as measured as the increase in daily infusions and escalation ratio. [*** indicates group x session interaction *p* < .01]﻿. **f** AIC lesions did not influence the acquisition and maintenance of cocaine self-administration under 1-h-short access conditions

Following surgery, sham lesioned animals showed a temporary decrease in cocaine intake that quickly returned to pre-surgery levels and showed further escalation after a week of daily extended access to the drug. In contrast, bilateral AIC-lesioned rats did not resume escalation of their daily intake. Instead, they acquired an allostatic titration level, which we have shown previously to characterise the maintenance of control over intake in rats given extended access to drugs (McNamara et al. 2010), thereby suggesting a restoration of control over intake [main effect of group x pre- and post-surgery block interaction *F*_1,12_ = 7.96, *p* < .02, pη^2^ = .398] (Fig. 3a).

Bilateral AIC lesions had no effect on the decrease of instrumental responding over a 90-min-long extinction session [main effect of group *F*_12,12_ = 1.27, NS, time *F*_17,204_ = 7.44, *p* < .0 01, pη^2^ = .382 and group x time interaction *F*_17,204_ < 1], both groups reaching as low as less than 0.4 lever presses per minute over the last 15 min of the session (Fig. 3b). Similarly, bilateral AIC lesions did not influence CS-induced reinstatement [main effect of group *F*_12,12_ = 1.27, NS and group x block interaction *F*_12,12_ = 1.08, NS]. In marked contrast, AIC lesions prevented the dose-dependent cocaine-induced reinstatement of instrumental responding [main effect of block *F*_3,36_ = 7.59, *p* < .001, pη^2^ = .387 and group x block interaction *F*_3,36_ = 3.36, *p* < .03, pη^2^ = .218] (Fig. 3b). Thus, whilst sham rats showed a dose-dependent increase in instrumental responding under extinction conditions in the first 15 min following non-contingent infusions of increasing doses of cocaine, displaying active lever press levels different from extinction at the dose of 1.6 mg/kg, AIC-lesioned rats did not show any statistically significant increase in responding. Thus, active lever presses by AIC-lesioned rats during the first 15 min following each non-contingent infusion never differed from extinction levels and were overall lower than those displayed by controls, a difference that was significant at the highest dose [*p* < .001] (Fig. 3b). Thus, cocaine, but not CS-induced reinstatement of instrumental responding, was impaired in AIC-lesioned rats.

Since a high-anxiety trait, as measured in the elevated plus maze, was previously shown to predict an increased propensity to escalate cocaine self-administration (Dilleen et al. 2012), we further tested, if bilateral AIC lesions in drug-naïve rats influenced anxiety. As illustrated in Fig. 3c, lesioned rats did not differ from their sham controls in the percentage of time spent or entries into the open arms of the EPM over the 5-min session [main effect of group *Fs*_1,15_ < 1, and group x time interaction *F* *s*_4,60_ < 1], i.e. showed no alteration in anxiety.

In marked contrast with post-training lesions, pre-training bilateral AIC lesions did not impair the escalation of cocaine intake, but instead resulted in a facilitation of loss of control. Thus, whilst extended access to cocaine resulted in a robust escalation of daily infusions overall [main effect of time *F*_18,360_ = 57.67, *p* < .001, pη^2^ = .742], escalation was much steeper in lesioned than that in sham rats [group x session interaction *F*_18,360_ = 5.42, *p* < .001, pη^2^ = .213] (Fig. 3d). This facilitated loss of control over cocaine intake by bilateral AIC lesions was further illustrated by the analysis of the escalation ratio which directly assesses how much increase in intake a rat displays on any given day as compared to the first day of extended access [main effect of group *F*_1,20_ = 6.08, *p* < .03, pη^2^ = .233 and group x session interaction *F*_17,340_ = 6.61, *p* < .001, pη^2^ = .248] (Fig. 3e). The facilitated escalation of cocaine intake observed following AIC lesions could not be accounted for by a difference in the reinforcing properties of cocaine as lesioned and sham rats similarly acquired cocaine self-administraton under short access conditions [main effect of group *F*_1,20_ < 1 and group x time interaction *F*_4,80_ < 1] (Fig. 3f).

## Discussion

The present data provide evidence that the AIC is involved in the regulation of cocaine self-administration and that its role dramatically changes during the different phases of the escalation of intake. Thus, in rats having escalated their intake of cocaine for several weeks, bilateral lesions of the AIC restored control and subsequently prevented drug-induced relapse. In marked contrast, similar lesions performed prior to drug exposure actually facilitated escalation of cocaine intake.

The disruptive effect of bilateral AIC lesions on escalated cocaine self-administration and the propensity to reinstate extinguished instrumental responding observed here are arguably consistent with previous human studies showing that craving correlates with activation of the insula and lesions of brain territories encompassing the insula result in a sudden loss of craving and decreased vulnerability to relapse (Naqvi et al. 2007). Since bilateral AIC lesions have been shown, both here (Fig. 2d, f) and previously (Belin-Rauscent et al. 2015; Cocker et al. 2016) not to induce a general motivational deficit, the present results provide further support for the hypothesis that the mechanisms originating in the AIC are causally involved in the rewarding properties of cocaine in chronic drug users that display diminished control over intake (Gray and Critchley 2007; Koob and Volkow 2016; Paulus and Stewart 2014).

Escalation of cocaine self-administration has been linked with an increased sensitivity to the motivational properties of cocaine (Ahmed and Koob 1998, 1999; Paterson and Markou 2003). Thus, one of the functions of the AIC, namely, tracking and updating the relative value of outcomes (Parkes and Balleine 2013; Parkes et al. 2015), may be compromised following AIC lesions. However, although this is consistent with the observed restoration of control over intake following AIC lesions in rats with a history of escalated cocaine self-administration, this interpretation is at odds with the finding that pre-training lesions do not prevent loss of control, but instead facilitate and exacerbate it. This suggests that instead of an involvement in mediating the incentive motivational properties of cocaine at early stages of extended access, the AIC eventually becomes engaged in mechanisms that functionally oppose those that initially support the development of escalation.

Thus, the opposite effects of AIC lesions, the directions of which are dependent on prior cocaine experience, are not readily encapsulated within a singular motivational construct. However, they seem to fit well within a framework of dynamic changes in interoceptive control over cocaine intake, which has been shown to occur in rats self-administering cocaine. Thus, rats will self-administer cocaine methiodide, a quaternary derivative of cocaine that does not cross the blood brain barrier, only if they have had previous experience of self-administering cocaine (Wang et al. 2013; Wise et al. 2008). This seminal observation revealed that the peripheral, interoceptive effects of cocaine, namely, increases in heart rate and blood pressure, that are initially aversive, become progressively associated with the subjective, presumably rewarding or reinforcing, effects of the drug (Williams and Barry 1966) over the course of cocaine self-administration, eventually becoming conditioned reinforcers in their own right (Everitt and Robbins 2016).

The AIC has been shown to be integral to interoceptive states associated with the anticipation or the manifestation of anxiety in humans (Adhikari 2014; Carlson et al. 2010; Critchley et al. 2004; Paulus and Stein 2006; Terasawa et al. 2013) and rats (Contreras et al. 2007; Li et al. 2014). However, AIC lesions did not alter the entries and time spent into the open arms of the elevated plus maze test. Thus, a generalised increase in anxiety, a behavioural marker of increased propensity to escalate cocaine intake (Dilleen et al. 2012), is unlikely to have contributed to the increase in drug intake following AIC lesions. Consequently, the facilitation of escalation of cocaine self-administration following pre-training AIC lesions reveals that, early after the introduction of extended access to the drug, the insula is mediating the aversive anxiogenic properties of cocaine (Ettenberg 2004; Ettenberg and Geist 1991; Guzman and Ettenberg 2007; Schank et al. 2008), which impair the development of escalation.

Chronic drug exposure, which is associated with anatomical and functional changes in the AIC (Luo et al. 2013; Mackey and Paulus 2013; Sinha 2011; Stewart et al. 2014), alters the perception of interoceptive cues (Wang et al. 2013; Wise et al. 2008), a process that may account for the observation that in the addicted state, the insula underlies the storage and recall of internal physiological states associated with the subjective rewarding effects of drugs (Craig 2009; Naqvi and Bechara 2009, 2010), promoting craving (Droutman et al. 2015). The present observation that post-training AIC lesions, shown to restore control over cocaine intake, also prevented subsequent cocaine-, but not CS-induced relapse is in agreement with a role of the insula in mediating interoceptive, but not exteroceptive, incentive-motivational, mechanisms in rats with a history of escalated cocaine self-administration.

Such a shift in the qualitative, but not quantitative, nature of insula-dependent interoceptive control over addictive behaviour is in agreement with evidence from recent pre-clinical studies showing that perturbations to AIC function following drug exposure led to decreased instrumental responding for drugs (Cosme et al. 2015; Pushparaj et al. 2015), whereas pre-training lesions exacerbated cocaine seeking (Pelloux et al. 2013). A qualitative, rather than quantitative understanding of the alteration in insula function during the progression from the initial drug use to addiction may also help reconcile the seemingly contradictory observations in humans that craving and relapse are associated with increased and decreased activity of the insula, respectively (Luo et al. 2013; Mackey and Paulus 2013; Naqvi and Bechara 2009; Sinha 2011; Stewart et al. 2014). Indeed, with reciprocal connections to the prefrontal cortex, striatum and amygdala (Allen et al. 1991), the AIC is in a unique position to differentially contribute to prefrontal cortex-dependent explicit motivational processes and executive control which are aberrantly engaged during craving (Naqvi and Bechara 2009; Noel et al. 2013) as well as mediating an interoceptive trigger of the amygdalo-striatal-dependent (Murray et al. 2015) maladaptive drug seeking habits that contribute to relapse (Everitt and Robbins 2005). However, which of these target regions mediate the insula’s interoceptive control over addictive behaviour remains to be determined.

What precipitates this qualitative shift in insula-mediated interoceptive mechanisms that contribute to the loss of control over cocaine intake is currently unknown. Consequently, one avenue for future research is to investigate neural and cellular adaptations downstream of the recruitment of brain stress neurochemical systems that have been shown to be necessary for the development and maintenance of escalation of cocaine self-administration (Koob 2009; Specio et al. 2008).

Overall, the present study demonstrates that the control exerted by the AIC over cocaine intake changes over the course of drug exposure that may reflect a change in the qualitative nature of the interoceptive control over behaviour. This resonates with the recent evidence that the AIC is a gateway from impulsivity to compulsivity (Belin-Rauscent et al. 2015), and therefore may represent an important neural component of addiction.
